# Gallbladder disease is associated with the risk of cardiovascular disease among Uyghurs in Xinjiang: a prospective cohort study

**DOI:** 10.1186/s12889-023-15098-9

**Published:** 2023-02-04

**Authors:** Rong Bai, Jiajia Wang, Jing Yang, Xiao Cheng, Shijie Zhang, Hongwei Zhang, Xiangwei Wu, Rulin Ma, Xianghui Zhang, Heng Guo, Xinyu Peng, Shuxia Guo

**Affiliations:** 1 Department of Hepatobiliary Surgery, The First Affiliated Hospital of Shihezi University School of Medicine, Shihezi, 832000 China; 2 Department of NHC Key Laboratory of Prevention and Treatment of Central, Asia High Incidence Diseases, The First Affiliated Hospital of Shihezi University School of Medicine, Shihezi, 832000 China; 3 Department of Public Health, Shihezi University School of Medicine, Shihezi, 832000 China

**Keywords:** Cardiovascular disease, Gallbladder disease, Cardiometabolic risk factors, Cohort study, Uyghur population

## Abstract

**Background:**

Gallbladder disease (GBD) can increase the risk of cardiovascular disease (CVD). However, GBD has rarely been reported in the less developed, rural areas of Xinjiang. This study aimed to determine the prevalence of GBD and incidence of CVD in a prospective cohort study in rural Xinjiang. Moreover, the study aimed to explore the association between GBD and CVD within this cohort.

**Methods:**

The study cohort included 11,444 Uyghur adults in Xinjiang, 3^rd^ division, from the 51st Mission. Study groups were classified according to whether GBD was present or absent at baseline. The occurrence of CVD was the end event. Demographic, anthropometric, and biochemical data were recorded, and the incidence of CVD in the GBD and non-GBD groups analysed. Cox proportional hazards regression models were used to assess the association between GBD and CVD and factors associated with their incidence. Several subgroup analyses were performed to assess CVD incidence in different subgroups. The interaction between GBD and cardiometabolic risk factors, and subsequent risk of developing CVD, was evaluated.

**Results:**

Prevalence of GBD in the study cohort was 10.29%. After a median follow-up of 4.92 years, the cumulative incidence of CVD in the study cohort was 10.49%, 8.43% in males and 12.65% in females. CVD incidence was higher in the GBD group (34.04% vs. 7.78%, HR = 4.96, 95% CI: 4.40–5.59). After multivariate adjustment, the risk of CVD remained higher in the GBD group (HR = 2.89, 95% CI: 2.54–3.29). Subgroup analyses showed male sex, smoking, alcohol consumption, lack of exercise, and abnormal renal function were all associated with increased risk of CVD. Moreover, the risk of CVD was markedly higher in GBD combined with cardiometabolic risk factors (hypertension, T2DM, dyslipidaemia, overweight, and abdominal obesity), than in cardiometabolic risk factors alone and this was higher in the GBD group than in the non-GBD group regardless of whether cardiometabolic risk factors were combined.

**Conclusion:**

GBD is an important independent risk factor for CVD development. Awareness of these associations will raise concerns among clinicians about the risk of cardiovascular disease in patients with GBD.

**Supplementary Information:**

The online version contains supplementary material available at 10.1186/s12889-023-15098-9

## Background

Common gallbladder diseases (GBD) include gallstone disease, cholecystitis, and gallbladder polyps. Long-term consumption of a diet high in protein, fat, and cholesterol can increase cholesterol in the bile, leading to supersaturation and gallstone formation. Increased cholesterol in the blood can be deposited within blood vessel walls. Over time, this results in the formation of atheromatous plaques. Existing studies show GBD to be an independent risk factor for cardiovascular disease (CVD), increasing the risk of CVD development [1–4]. Gallbladder polyps are mainly composed of cholesterol and inflammatory polyps, and their growth and development are closely related to cholesterol metabolism and inflammation [5]. Therefore, gallbladder polyps can initiate and advance development of CVD, whereas early treatment of gallbladder polyps may reduce the risk of CVD [6]. In addition, cholecystitis may induce CVD through both cardiac and cerebral hypoperfusion [7]. Moreover, existing studies report evidence suggesting cholecystectomy patients are at a high risk of developing CVD [8, 9]. The prevalence of GBD is as high as 8.80%-15.87% in inland cities in China [10, 11]; however, GBD has rarely been reported in rural areas of Xinjiang.

Risk factors for CVD include smoking, dyslipidaemia, obesity, metabolic syndrome, hypertension, insulin resistance, and diabetes, all of which are also commonly associated with the development of GBD [1–4]. Xinjiang is a multi-ethnic region; thus, unique dietary habits and lifestyles give rise to the development of a variety of diseases in the same region and among different ethnic groups. Epidemiological surveys have shown that the prevalence of gallbladder stones is higher among Uyghurs than among Han Chinese, with significant ethnic differences [12]. Moreover, previous studies have found the prevalence of metabolic syndrome [13], diabetes [14, 15]and obesity [15] is higher in the Uyghur population than in the Han Chinese population. As such, we therefore conducted a large 5-year cohort study aiming to determine the prevalence of GBD and onset of CVD in the Uyghur population in rural Xinjiang, China. Moreover, we aimed to explore the association between GBD and the risk of developing CVD.

## Materials and methods

### Study population

This cohort study used a typical sampling method, selecting Uyghur adults in Xinjiang, 3^rd^ division, from the 51st regiment of the farm from the Uyghur population. A baseline survey was conducted from August to September 2016, and three follow-up visits were conducted in 2019, 2020, and 2021. A total of 14,321 study participants were included at baseline, and 1527 participants were excluded due to incomplete information, severe illness, unconsciousness, inability to cooperate, unwillingness, pregnancy, and transient population. A further 1064 participants with existing CVD (ischaemic heart diseases (IHD), pulmonary embolism, cerebrovascular diseases, peripheral vascular disease, etc.) were also excluded. CVD survival time was defined as the follow-up time from August or September 2016 to either the first CVD event, or the end of follow-up. Written informed consent was obtained from all participants. The Institutional Ethics Review Board (IERB) of the First Hospital of Shihezi University School of Medicine approved this study (IERB number: SHZ2010LL01).

### Data collection

All study participants underwent a face-to-face questionnaire survey to collate demographic information, and the questionnaire content remained consistent at both baseline and post-follow-up periods. An epidemiological survey, conducted by trained professional investigators collected details including personal and family history, disease history, lifestyle, height, weight, waist circumference (WC), body mass index (BMI), systolic blood pressure (SBP), diastolic blood pressure (DBP), presence of existing type 2 diabetes mellitus (T2DM) and presence of existing hypertension. Serum total bilirubin (TBIL), direct bilirubin (DBIL), indirect bilirubin (IBIL), fasting blood glucose (FBG), triglycerides (TG), total cholesterol (TC), high-density lipoprotein cholesterol (HDL-C), low-density lipoprotein cholesterol (LDL-C), serum creatinine (SCr), and serum uric acid (UA) levels were all measured using an automated biochemistry analyser (Olympus Au 2700; Olympus Diagnostics, Hamburg, Germany). Accuracy and completeness of all information was ensured by collecting both hospital and medical insurance records of each participant.

Smoking was defined as smoking ≥ 1 cigarette per day for ≥ 6 months [16]. Drinking was defined as alcohol consumption ≥ 1 day per week for ≥ 6 months [17]. Exercise was defined as ≥ 1 exercise session per week, lasting ≥ 30 min each. Electrocardiography (ECG) abnormalities were assessed based on 12-lead ECG examination, medical insurance and/or hospital records. Abnormalities included arrhythmia, AV block, bundle branch block, premature ventricular beats, premature atrial beats, tachycardia, bradycardia, atrial hypertrophy, ST-T changes, and T-wave changes without a diagnosis of CVD. Hypertension was defined as SBP ≥ 140 mmHg and/or DBP ≥ 90 mmHg on physical examination, medical insurance and/or hospital records, or current use of antihypertensive medication [18]. T2DM was defined as fasting blood glucose ≥ 7.0 mmol/L on physical examination, medical insurance and/or hospital records, or currently taking medication to control blood glucose levels [19]. Hyperbilirubinemia was defined as serum TBIL > 17.10 μmol/L [20]. Dyslipidaemia was defined based on TC ≥ 6.20 mmol/L, TG ≥ 2.30 mmol/L, LDL-C ≥ 4.10 mmol/L, HDL-C < 1.00 mmol/L, existing dyslipidaemia diagnosis, and current statin use [21]. BMI = weight/height^2^ (kg/m^2^), where 18.50–23.90 kg/m^2^ indicates a normal weight; 24.00–27.90 kg/m^2^, overweight; and ≥ 28.00 kg/m^2^, obesity [22]. Here, we refer to BMI ≥ 24.00 kg/m^2^ as overweight, and abdominal obesity was defined as WC > 90 cm for males and > 80 cm for females [22]. With reference to the Guidelines for the Prevention and Treatment of Dyslipidaemia in Chinese Adults (2016) [21]: TC < 5.20 mmol/L, TG < 1.70 mmol/L, LDL-C < 3.40 mmol, and HDL-C ≤ 1.00 mmol were defined as the low-level group, and TC ≥ 5.20 mmol/L, TG ≥ 1.70 mmol/L, LDL-C ≥ 3.40 mmol/L, and HDL-C > 1.00 mmol/L as the high-level group. UA < 404.60 µmol/L was defined as the low-level group, and UA ≥ 404.60 µmol/L was defined as the high-level group [23, 24]. According to the CKD-EPI formula [25]:

In males: 1) SCr ≤ 0.9 mg/dL, estimated glomerular filtration rate (eGFR) = 141 x (SCr (mg/dL)/0.9)^−0.411^ × (0.993)^age^; 2) SCr > 0.9 mg/dl, eGFR = 141 × (SCr (mg/dl)/0.9)^−1.209^ × (0.993)^age^. In females: 1) SCr ≤ 0.7 mg/dL, eGFR = 141 × (SCr (mg/dL)/0.7)^−0.329^ × (0.993)^age^; 2) SCr > 0.7 mg/dL, eGFR = 141 × (SCr (mg/dL)/0.9)^−1.209^ × (0.993)^age^. SCr unit conversion: 1 mg/dL = 88.4 μmol/L. Abnormal renal function was defined as eGFR < 90 mL/min/1.73 m^2^, and eGFR ≥ 90 mL/min/1.73 m^2^) indicated normal renal function. Abdominal ultrasound and electrocardiography were performed by specialist clinicians.

### Diagnostic criteria for CVD

Diagnosis of a new CVD event was made if any of the following criteria for CVD diagnosis was met, as recommended by the World Health Organization's International Classification of Diseases, 10th Revision (ICD10)[26]: IHD (ICD10: I20-I25), pulmonary embolism (ICD10: I26), cerebrovascular diseases (ICD10: I60-I69), peripheral vascular disease (ICD10: I70-I82) or death due to the above causes. If the same type of event occurred twice or more in the same participant, the first event was used as the end event. The above information was obtained from the participant self-reported questionnaire (which included proof of clinical diagnosis), and inpatient medical records.

### Diagnostic criteria for GBD

Diagnosis of GBD was made when any of the following diagnostic criteria of the World Health Organization's International Classification of Diseases, 10th Revision (ICD10) [26, 27] were met: cholelithiasis (ICD10:K80), cholecystitis (ICD10:K81), malignant neoplasm of the gallbladder (ICD10:C23), cholangitis (ICD10:K83.0), and other GBDs (ICD10: K82.0-K82.4, K82.8). The other GBDs included gallbladder obstruction, gallbladder hydrops, gallbladder perforation, gallbladder fistula, gallbladder cholesterolosis, gallbladder polyp, gallbladder hypertrophy, and cholecystectomy. In addition, in this study we also included cholecystectomy (ICD10:0FT44ZZ, 0FB44ZZ,0FB48ZZ, 0FB40ZZ, 0FB43ZZ, 0FT40ZZ), with diagnosis confirmed by ultrasound examination by a specialist sonographer or a participant self-reported questionnaire (which included proof of clinical diagnosis), and inpatient medical records.

### Statistical analysis

Continuous variables were expressed as mean ± standard deviation, and percentages were used to describe categorical variables. CVD event rate was calculated as the number of events per 10,000 people, per year of follow-up. The chi-square test was used to compare categorical variables, and the Mann–Whitney U test was used to compare differences between groups in continuous variables. The Kaplan–Meier method was used to estimate the cumulative incidence of CVD events. Hazard ratios (HRs) and 95% confidence intervals (CIs) for CVD occurrence were estimated using the Cox hazards proportional model. A multivariable model for CVD was constructed using stepwise regression. This included variables significantly associated with the occurrence of CVD in univariate analysis and known traditional risk factors for CVD. A likelihood ratio test was used to determine whether the added variables significantly improved the model using a significance threshold of *P* < 0.05. The model was eventually adjusted for sex, age, exercise, hypertension, T2DM, overweight, and HDL levels. Analyses were performed using SPSS 25 (SPSS Inc., Chicago, IL, USA), R version 4.2.0 (R Foundation for Statistical Computing, Vienna, Austria), and graphs were drawn using Office and R 4.2.0, with *P* < 0.05 (two-tailed test) indicating statistical significance.

The risk of CVD was assessed by subgroup analysis of sex, age (< 35 and ≥ 35 years), smoking, drinking, exercise, eGFR group, and TG, TC, LDL, and HDL levels. In addition, the interaction between GBD and cardiometabolic-related factors including hypertension, T2DM, dyslipidaemia, overweight, and central obesity, on the risk of CVD was analysed.

## Results

### Baseline characteristics

**Fig. 1:**
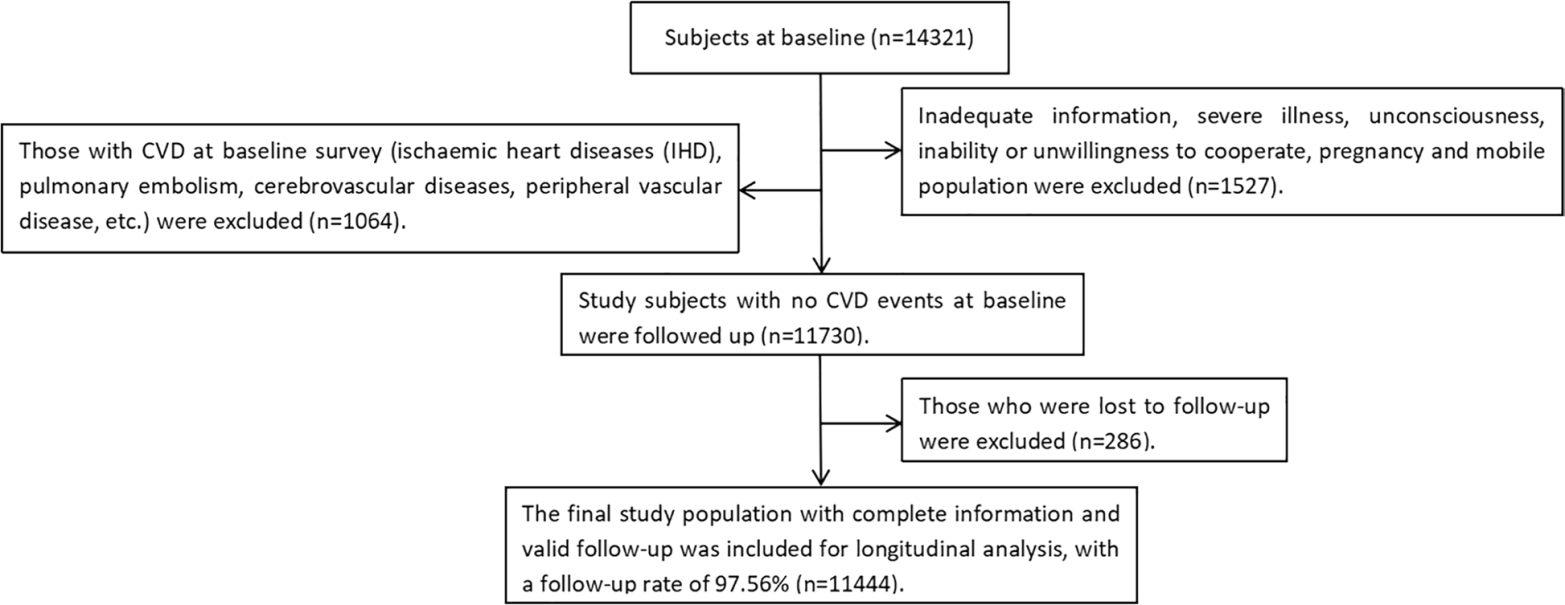
Flow chart of the study cohort including inclusion and exclusion criteria. (CVD, cardiovascular disease)

The final cohort consisted of 11,444 study participants, included in the longitudinal analysis; the follow-up rate was 97.56% (Fig. 1). The baseline GBD group consisted of 1178 participants, of whom there were 717 with cholecystitis, 313 with cholelithiasis, 86 with cholecystectomy, 37 with gallbladder polyps, 9 with gallbladder hypertrophy, 7 with gallbladder cholesterolosis, 3 with gallbladder fistula, 2 with cholangitis, 1 with gallbladder malignancy, 1 with gallbladder obstruction, 1 with gallbladder hydrops, and 1 with gallbladder perforation. The baseline GBD prevalence was 10.29%; 6.04% in males and 14.77% in females. The mean age of this cohort was 36.56 ± 13.49 years, and the mean age of the GBD group was significantly higher than that of the non-GBD group (*p* < 0.05). The proportion of participants with ECG abnormalities and a family history of CVD was also higher in the GBD group than in the non-GBD group (all *p* < 0.05). In the GBD group, prevalence of hypertension was 40.49%; T2MD, 11.38%; dyslipidaemia, 39.90%; overweight, 74.53%; and abdominal obesity, 84.80%. All of these rates were significantly higher than those in the non-GBD group (all *p* < 0.05). Compared to the non-GBD group, individuals with hypertension, T2DM, overweight, abdominal obesity, high levels of TG (TG ≥ 1.70 mmol/L) and TC (TC ≥ 5.20 mmol/L) were more likely to have GBD. However, there were no significant differences between the two groups in hyperbilirubinemia, UA levels, FBG, and DBIL (all *p* > 0.05). Both GBD and Non-GBD groups showed that age, exercise, hypertension, T2DM, overweight, abdominal obesity and TG levels were associated with increased CVD risk (all *p* < 0.05) (Table 1 and Supplementary Table S1).

### CVD incidence and related factors

**Fig. 2:**
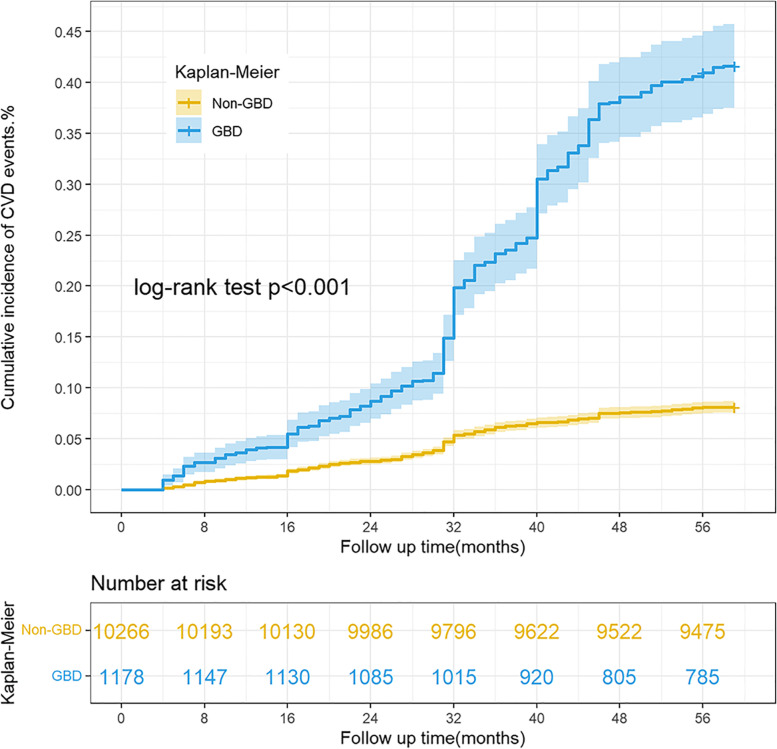
Kaplan–Meier curve: cumulative incidence of CVD based on GBD subgroups. (CVD, cardiovascular disease**;** GBD, gallbladder disease)

After a median follow-up of 4.92 years, 1200 study subjects had a new CVD event with a cumulative incidence of 10.49%, 8.43% for men and 12.65% for women (Table 1 and Supplementary Table S1). The cumulative incidence of CVD in the GBD group was 34.04%, which was significantly higher than that in the non-GBD group at 7.78% (HR = 4.96, 95% CI: 4.40–5.59) (Table 2 and Fig. 2).

Sex, age, smoking, drinking, exercise, hypertension, T2DM, dyslipidaemia, overweight, abdominal obesity, TG levels, TC levels, LDL levels, and HDL levels were all strongly associated with CVD by univariate Cox regression analysis (*P* < 0.05). Further stepwise forward regression analysis using Cox regression analysis showed that sex, age, exercise, hypertension, T2DM, overweight, and HDL levels all independently influenced the occurrence of CVD (*p *< 0.05) (Supplementary Figure S1). After the multivariate adjustment, the risk of developing CVD was still higher in the GBD group than in the non-GBD group (HR = 2.89, 95% CI: 2.54–3.29) (Table 2).

Model a was unadjusted. Model b was adjusted for age and sex. Model c was further adjusted for exercise, hypertension, T2DM, overweight, and HDL levels. * Rate per 10,000 person-years. (GBD, gallbladder disease; T2DM, type 2 diabetes mellitus; CVD, cardiovascular disease; HDL, high-density lipoprotein cholesterol).

### Effect of subgroup analysis and interaction between influencing factors on the risk of developing CVD

**Fig. 3:**
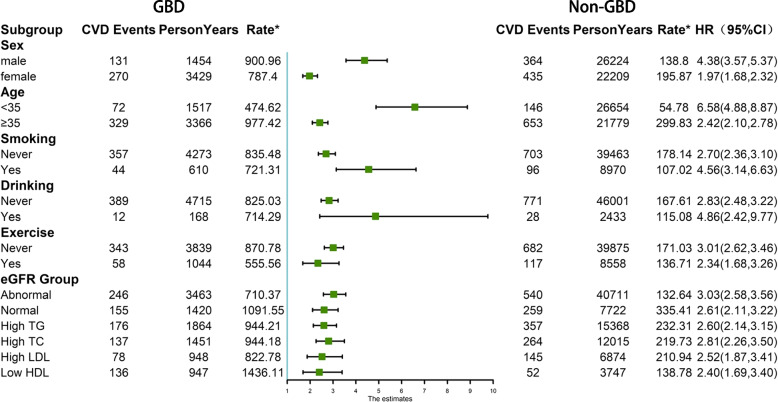
Subgroup analysis of the risk of CVD in different groups. All models were adjusted for sex, age, exercise, hypertension, T2DM, overweight, and HDL levels. *Rate per 10,000 person-years. (GBD, gallbladder disease; CVD, cardiovascular disease; eGFR, estimated glomerular filtration rate**;** TG, triglyceride; TC, total cholesterol; LDL, low-density lipoprotein cholesterol; HDL, high-density lipoprotein cholesterol)

Subgroup analysis by sex, age, smoking, drinking, exercise, eGFR, and TG, TC, LDL, and HDL levels, showed that the risk of CVD was significantly higher in the GBD group than in the non-GBD group in all subgroups. Moreover, being male, smoking, drinking, lack of exercise, and abnormal renal function also increased the likelihood of a CVD event, and high levels of TG, TC, and LDL, and low levels of HDL increased the risk of CVD (Fig. 3). Assessment of the risk of CVD from the interaction of GBD and cardiometabolic risk factors (hypertension, T2DM, dyslipidaemia, overweight, and abdominal obesity) showed the risk was markedly higher in GBD combined with cardiometabolic risk factors, than in cardiometabolic risk factors alone. Moreover, this was higher in the GBD group than in the non-GBD group regardless of whether cardiometabolic risk factors were combined (Table 3 and Supplementary Figure S2).

## Discussion

In this prospective cohort study, the prevalence of GBD among Uyghur adults was 10.29%, 6.04% in males and 14.77% in females. The cumulative incidence of CVD was 10.49%, 8.43% in males and 12.65% in females. Results suggest GBD significantly increases the risk of CVD (HR = 2.89, 95% CI: 2.54–3.29). Moreover, GBD remains strongly associated with the risk of CVD after subgroup analysis of traditional CVD risk factors, and analysis of the interaction between GBD and cardiometabolic-related risk factors. Thus, this study provides strong evidence that GBD is an independent risk factor for CVD.

In a 2018 cross-sectional study of adults in Jilin Province, Northeast China, the prevalence of GBD was 8.8% [10]. Prevalence of GBD in our study was slightly higher, 10.29%. This difference could be due to only cholecystitis and gallbladder stones being the included GBD types in the Jilin study. In this study, a wider range of definitions of GBD disease types were used. However, this difference may also be due to the Uyghur population living in Xinjiang, a region with high latitudes and long winters. The Uyghur population also consume fewer vegetables and fruits, preferring meats such as beef and lamb. This is significant, as a non-vegetarian diet can increase the risk of GBD [28]. A retrospective epidemiological survey involving 30,901 people in Songjiang, Shanghai showed a prevalence of 15.87% for benign GBD [11]. The definition of GBD in this study was similar to that in our study, including gallbladder stones, gallbladder polyps, and cholecystitis; however, prevalence was higher than in the rural population in this study (10.29%). This may be explained by the high standard of living in Shanghai. Shanghai is one of the core cities of China's metropolis, where fast-paced living leads to irregular eating patterns. Examples include skipping meals and overeating, both of which are likely to accelerate the onset of GBD. In addition, our study showed that the prevalence of GBD is more than twice as high in women as in men (14.77% vs. 6.04%), comparable to the prevalence of 12.8% in women and 4.4% in men reported in the study in Jilin Province [10]. Similar findings were obtained in a survey of gallstones in a white population in the United States (prevalence was 16.6% in women and 8.6% in men) [29, 30]. This suggests that sex differences in the occurrence of GBD have little to do with race and geography. In developed females, oestrogen can promote cholesterol secretion by the liver. This can result in oversaturation of bile with cholesterol and induce gallstone disease [31]. Moreover, Xinjiang Uyghur women have a high fertility rate [32], and it is shown that a history of multiple births can also increase the risk of GBD [33]. Our findings focus on Uyghur populations in the rural areas of Xinjiang, China. Despite this, the findings remain important as a reference for studies of GBD in other regions of China.

The present study found a CVD incidence of 10.49% in the rural Uyghur population in Xinjiang, which is generally higher than the current CVD incidence in inland cities in China, 2.28%–9.09% [34–36]. This may be attributed to the predominantly salty and oily dietary preferences, coupled with a low intake of vegetables and fruits in the Uyghur population. This also leads to a high prevalence of obesity [15] and metabolic syndrome [13], both of which are risk factors for CVD and, can contribute to the occurrence of CVD events [37]. In addition, the Uyghur diet is higher in saturated fatty acids and trans-fatty acids, which can lead to elevated LDL concentrations and increased atherosclerotic plaque formation, which can contribute to CVD events [38, 39]. Specific genetic loci, such as the rs429358 polymorphism in apolipoprotein E, have been suggested to be closely associated with the development of coronary heart disease in Xinjiang Uyghurs [40]. As such, all these factors may have contributed to the high incidence of CVD in Uyghurs. In our study, the incidence of CVD was significantly higher in the GBD group than in the non-GBD group. Even after adjusting for the confounding factors, the risk of CVD was 2.89 times greater in the GBD group than in the non-GBD group, and a strong association between GBD and CVD remained. Numerous studies have reported a strong relationship between gallstones, gallbladder polyps, cholecystectomy, cholecystitis, and other gallbladder diseases and cardiovascular diseases. In a cohort study in the United States involving 270,000 people in three different populations, all three cohorts showed that those with a history of gallstones had a higher risk of coronary heart disease (CHD), compared with those without a history of gallstones [3]. A similar finding was made in a large prospective study in China covering ages 30–79 years. The study reported that after a median follow-up of 7.2 years, the risk of IHD was 1.23 times greater in those with a history of gallstones than in those without [41]. A retrospective cohort study of 19,612 adults in Korea showed that the risk of IHD in the gallbladder polyp group was 1.43 times higher than that in the non-gallbladder polyp group [6]. A prospective cohort study of 2815 adults over 20 years of age in Taiwan, China, similarly concluded that gallbladder polyps increased the risk of CHD. However, the study also showed no significant association between cholecystectomy and the development of CHD [42], though others have argued that cholecystectomy is strongly associated with CVD. A large multi-centre EPIC cohort study in Germany reported that patients who underwent cholecystectomy had a 1.32 times higher risk of CVD events than those who did not undergo cholecystectomy [4]. Ruhl et al. [43] and Fan et al. [44] similarly concluded that cholecystectomy may be responsible for an increased risk of CVD and is an independent factor influencing CVD. Regarding the relationship between cholecystitis and CVD, a specific link between cholecystitis or biliary colic and angina pectoris, arrhythmias, and non-specific ST-T waves was proposed as early as 1986 [45]. Subsequent clinical cases have reported elevated troponin and ECG-like signs of myocardial ischaemia (T-wave inversion or ST-segment depression) in patients with acute cholecystitis. This further suggests a close association between both GBD and CVD [46, 47]. Previous studies have shown that GBDs such as cholelithiasis, gallbladder polyps, cholecystectomy, and cholecystitis all increase the risk of CVD to varying degrees. This would explain why the GBD group in our study had a higher risk of CVD than these individual factors mentioned above. Our study is the first to report an association between GBD and CVD in the Xinjiang Uyghur population, where genetic variation is little due to low population mobility.

Our subgroup analysis found a higher risk of CVD was still observed in the GBD group, suggesting that our model is valid. The results of this study also showed that males, smokers, alcohol drinkers, those with little physical inactivity, and people with GBD with abnormal kidney function, are more likely to have a CVD event. Given that smoking, drinking, and lack of exercise are socially accepted risk factors for chronic diseases, these are not repeated here. Although the prevalence of GBD in females was more than twice that of males in this study, the risk of CVD was much higher in males with GBD than in females. This may be due to men making up a greater proportion of those with poor lifestyle habits, such as smoking and alcohol consumption, thus being more likely to be exposed to risk factors for CVD. Oestrogen in women stimulates the uptake of LDL by the liver and inhibits the metabolism of HDL, which is largely cardioprotective. Men are also more likely to have accumulation of visceral fat owing to differences in fat distribution between the sexes, and are therefore at a higher risk of CVD [48]. Cardiometabolic risk factors such as hypertension, T2DM, dyslipidaemia, overweight, and abdominal obesity may not only contribute to CVD when present individually, but their different aggregation characteristics may also increase the risk of CVD. Our study confirmed this finding, showing that the combination of GBD with cardiometabolic risk factors significantly increases CVD risk. Furthermore, a significant increase in CVD risk with GBD was seen in groups without hypertension, T2DM, dyslipidaemia, overweight, or abdominal obesity. Hence, the GBD group remained at a higher risk of CVD regardless of the presence or absence of cardiometabolic risk factors. In light of this, increased monitoring of GBD should be considered in addition to cardiometabolic risk factors, which may indicate CVD occurrence.

Whilst the present study suggests that GBD is an independent risk factor for the development of CVD, the pathogenesis of CVD due to GBD is unclear. Cholesterol metabolism may be involved with both gallstones and gallbladder polyps being closely associated with increased cholesterol secretion. Cholesterol accumulation is one of the main common pathophysiological mechanisms of gallstone disease and CVD. In patients with gallstones, bile acid secretion decreases, whereas cholesterol secretion continues to increase. When the vascular endothelium is damaged, oxidised LDL cholesterol is deposited into the vascular wall and phagocytosed by macrophages in the blood vessels, forming foam cells, which then cause a series of inflammatory reactions, fibrosis, proliferation of smooth muscle cells, and formation of atheromatous plaques which can be deposited in the vascular wall [3, 49]. However, acetyl coenzyme A acetyltransferase 2 (ACAT2) promotes both cytokinesis of cholesterol in the mucosa of gallbladder polyps and the binding of cholesteryl esters to lipoproteins. This increases the secretion of very low-density lipoproteins (VLDL) [50, 51], which further promotes atheromatous plaque formation. Over time, atheromatous plaques eventually lead to atherosclerotic stenosis, which is one of the main causes of CVD. Bile-heart reflex is the second pathophysiological mechanism proposed in the pathogenesis of GBD and CVD. Both the gallbladder and heart have vagus nerve branches. Gallbladder inflammation or bile duct dilatation can cause the vagus nerve to be repeatedly and abnormally excited for a prolonged period. This can reflexively cause the heart rate to slow and the coronary arteries to spasm, resulting in insufficient blood supply to the heart and aggravating the development of CVD [46].

The large prospective cohort study design, is one of the strengths of this study, facilitating exploration of the time-dependent relationship between GBD and CVD. In addition, this study is the first investigation into the relation between GBD and CVD in the Xinjiang Uyghur population, a multi-ethnic region with unique dietary habits and lifestyles. However, there are many limitations to this study. Firstly, the relatively broad definition of GBD fails to separately analyse the risk of gallstone disease, cholecystitis, gallbladder polyps, and cholecystectomy in relation to the development of CVD. Secondly, the study focuses on the Uyghur population in rural areas of Xinjiang; as such, the findings may not be applicable to other groups given differences in genetics, diet, and living practices. Additionally, family history of GBD was not collated. Moreover, the influence of genetic factors on GBD and CVD was not assessed. Finally, the study model used was not externally validated, and its predictive power has yet to be further assessed.

## Conclusions

In conclusion, our study showed that GBD is an important and independent risk factor for the development of CVD. As such, clinicians should be aware of the importance of appropriately detecting patients with gallbladder disease, identifying high-risk CVD in a timely manner, and taking measures to stratify management, which provides another way of thinking about precision medicine.

**Table 1:** Baseline characteristics of the study population with a CVD event in the GBD subgroup

Variable	GBD		(χ2/Z)_1_	*P *_*1*_	Non-GBD		(χ2/Z)_2_	*P*_*2*_
	**CVD**	**Non-CVD**			**CVD**	**Non-CVD**		
N (%)	401(34.04)	777(66.96)			799(7.78)	9467(92.22)		
Age (years)	48.70 ± 13.78	41.49 ± 13.04	-8.49	< 0.001	47.73 ± 13.79	34.70 ± 12.65	-24.76	< 0.001
Male	131(32.67)	224(28.83)	1.85	0.174	364(45.56)	5154(54.44)	23.40	< 0.001
Smoking (%)	44(10.97)	98(12.61)	0.67	0.413	96(12.02)	1774(18.74)	22.36	< 0.001
Drinking (%)	12(3.99)	27(3.47)	0.19	0.661	28(3.50)	481(5.08)	3.89	0.049
Exercise (%)	58(14.46)	181(23.29)	12.75	< 0.001	117(14.64)	1690(17.85)	5.23	0.022
ECG abnormality (%)	126(31.42)	286(36.81)	3.38	0.066	231(28.91)	2652(28.01)	0.29	0.588
Hypertension (%)	251(62.59)	226(29.09)	123.24	< 0.001	385(48.19)	2431(25.68)	187.50	< 0.001
T2DM (%)	64(15.96)	70(9.01)	12.68	< 0.001	118(4.77)	420(4.44)	158.39	< 0.001
Hyperbilirubinemia (%)	25(6.23)	65(8.37)	1.7	0.192	66(8.26)	808(8.53)	0.07	0.789
Dyslipidaemia (%)	17(4.24)	41(5.280)	0.61	0.436	89(11.14)	3273(34.57)	183.72	< 0.001
Overweight (%)	324(80.80)	554(71.30)	12.57	< 0.001	471(58.95)	5361(56.63)	111.79	< 0.001
Abdominal obesity (%)	361(90.02)	638(82.11)	12.86	0.001	698(87.36)	6760(71.41)	94.37	< 0.001
Family history of CVD (%)	78(19.45)	170(21.88)	0.94	0.333	118(14.77)	1403(14.92)	0.00	0.969
TG levels (mmol/L, %)			5.77	0.016			60.83	< 0.001
Low (< 1.70)	225(56.11)	492(63.32)			442(55.32)	6509(68.75)		
High (≥ 1.70)	176(43.89)	285(36.68)			357(44.68)	2958(31.25)		
TC levels (mmol/L, %)			4.69	0.3			29.28	< 0.001
Low (< 5.20)	264(65.84)	559(71.94)			535(66.96)	7157(75.60)		
High (≥ 5.20)	137(34.16)	218(28.06)			264(33.04)	2310(24.40)		
LDL levels (mmol/L, %)			0.05	0.826			10.35	0.001
Low (< 3.40)	323(80.55)	630(81.08)			654(81.85)	8142(86.00)		
High (≥ 3.40)	78(19.45)	147(18.92)			145(18.15)	1325(14.00)		
HDLlevels (mmol/L, %)			56.7	< 0.001			1.72	0.190
Low (≤ 1.00)	136(33.92)	116(14.93)			52(6.51)	738(7.80)		
High (> 1.00)	265(66.08)	661(85.07)			747(93.49)	8729(92.20)		
UA levels (μmol/L, %)			2.87	0.9			0.00	0.968
Low (< 404.60)	384(95.76)	758(97.55)			775(97.00)	9185(97.02)		
High (≥ 404.60)	17(4.24)	19(2.45)			24(3.00)	282(2.98)		
WC (cm)	96.34 ± 12.33	93.10 ± 13.01	-4.47	< 0.001	96.72 ± 14.00	89.60 ± 12.95	-14.23	< 0.001
FBG (mmol/l)	5.40 ± 4.35	4.88 ± 1.76	-4.26	< 0.001	5.30 ± 2.61	4.88 ± 1.52	-1.99	0.047
BMI (kg/m^2^)	27.83 ± 4.87	26.84 ± 4.77	-3.71	< 0.001	27.46 ± 4.80	25.28 ± 4.47	-13.07	< 0.001
TG (mmol/L)	1.98 ± 1.49	1.72 ± 1.26	-3.12	0.002	1.95 ± 1.46	1.63 ± 1.23	-7.96	< 0.001
TC (mmol/L)	4.83 ± 1.06	4.65 ± 1.19	-3.09	0.002	4.79 ± 1.06	4.64 ± 2.00	-6.41	< 0.001
UA (μmol/L)	262.30 ± 74.06	249.43 ± 72.90	-2.92	0.004	265.78 ± 70.24	256.06 ± 70.53	-3.74	0.004
SCr (μmol/L)	70.10 ± 15.61	67.69 ± 14.75	-2.19	0.029	70.93 ± 15.52	72.27 ± 14.34	-2.61	0.009
TBIL (μmol/L)	9.58 ± 5.02	10.80 ± 6.28	-4.11	< 0.001	10.63 ± 6.34	10.58 ± 6.12	-0.09	0.930
IBIL (μmol/L)	6.41 ± 4.80	6.68 ± 6.19	-0.43	0.665	6.94 ± 6.27	6.23 ± 5.64	-3.04	0.002
DBIL (μmol/L)	4.39 ± 3.46	4.51 ± 2.08	-1.52	0.129	4.32 ± 2.07	4.57 ± 2.37	-3.13	0.002
LDL-c (mmol/L)	2.72 ± 0.74	2.68 ± 0.81	-0.6	0.547	4.32 ± 2.07	2.59 ± 0.74	-4.47	< 0.001
HDL-c (mmol/L)	1.49 ± 0.53	1.47 ± 0.46	-0.51	0.612	1.44 ± 0.51	1.57 ± 0.55	-9.36	< 0.001
SBP (mmHg)	139.77 ± 19.95	127.44 ± 17.07	-10.49	< 0.001	138.53 ± 24.16	124.94 ± 17.00	-15.77	< 0.001
DBP (mmHg)	79.52 ± 13.28	75.16 ± 11.54	-5.57	< 0.001	79.45 ± 13.87	73.45 ± 11.72	-11.92	< 0.001
eGFR (ml/min/1.73 m^2^)	95.60 ± 17.45	102.01 ± 17.84	-5.96	< 0.001	97.56 ± 17.91	107.20 ± 17.83	-15.69	< 0.001

Values are presented as mean ± standard deviation or n (%). P_1_ = results of the chi-square or Mann–Whitney U-test for differences in baseline parameters of the participants with GBD between the CVD and non-CVD groups; P_2_ = results of the chi-square or Mann–Whitney U-test for differences in baseline parameters of the participants with non-GBD between the CVD and non-CVD groups

(GBD gallbladder disease, ECG electrocardiography, T2DM type 2 diabetes mellitus, TG triglyceride, TC total cholesterol, LDL low-density lipoprotein cholesterol, HDL high-density lipoprotein cholesterol, UA uric acid, WC waist circumference, FBG fasting blood glucose, BMI body mass index, SCr serum creatinine, TBIL serum total bilirubin, IBIL indirect bilirubin, DBIL direct bilirubin, SBP systolic blood pressure, DBP diastolic blood pressure, eGFR estimated glomerular filtration rate, CVD cardiovascular disease)

**Table 2:** Cox regression model for the relationship between GBD and CVD

**Group**	**CVD, n (%)**	**Person-Years**	**Rate**^d^	**HR**^**a**^**(95%CI)**	**HR**^**b**^**(95%CI)**	**HR**^**c**^**(95%CI)**
Non-GBD	799(7.78)	48,433	164.97	1.00 (ref.)	1.00 (ref.)	1.00 (ref.)
GBD	401(34.04)	4883	821.22	4.96(4.40,5.59)	3.16(2.78,3.59)	2.89(2.54,3.29)

Model a was unadjusted

Model b was adjusted for age and sex

Model c was further adjusted for exercise, hypertension, T2DM, overweight, and HDL levels

^d^ Rate per 10,000 person-years. (*GBD* gallbladder disease, *T2DM* type 2 diabetes mellitus, *CVD* cardiovascular disease, *HDL* high-density lipoprotein cholesterol)

**Table 3:** Analysis of the interaction of GBD and cardiometabolic risk factors associated with CVD

Subgroup		CVD Events	Person-Years	Rate^d^	HR^a^(95%CI)	HR^b^(95%CI)	HR^c^(95%CI)
GBD	Hypertension						
-	-	414	35,591	116.32	1.00 (reference)	1.00 (ref.)	1.00 (ref.)
-	+	385	12,842	299.8	2.58 (2.25,2.97)	1.89 (1.64,2.18)	1.82 (1.58,2.11)
+	-	150	3093	484.97	4.17 (3.46,5.02)	2.76 (2.28,3,35)	2.65 (2.19,3.23)
+	+	251	1790	1402.23	11.80 (10.08,13.81)	6.02( 5.09,7.12)	5.58 (4.71,6.62)
GBD	T2DM						
-	-	681	46,083	147.78	1.00 (reference)	1.00 (ref.)	1.00 (ref.)
-	+	118	2350	502.13	3.40(2.79,4.13)	2.28 (1.87,2.77)	2.12 (1.74,2.58)
+	-	337	4380	769.41	5.15(4.52,5.87)	3.39 (2.96,3.89)	3.21 (2.80,3.69)
+	+	64	503	1272.37	8.54(6.61,11.03)	3.94 (3.03,5.140	3.50 (2.68,4.56)
GBD	Dyslipidemia						
-	-	474	34,761	136.36	1.00 (reference)	1.00 (ref.)	1.00 (ref.)
-	+	325	13,672	237.12	1.74 (1.51,2.00)	1.34 (1.16,1.55)	1.10 (0.94,1.28)
+	-	224	2993	748.41	5.43 (4.63,6.36)	3.31 (2.81,3.91)	3.00 (2.54,3.56)
+	+	177	1890	936.51	6.81 (5.73,8.10)	3.80 (3.18,4.54)	2.98 (2.46,3.60)
GBD	Overweight						
-	-	193	20,661	93.41	1.00 (reference)	1.00 (ref.)	1.00 (ref.)
-	+	606	27,772	218.21	2.34 (1.99,2.75)	1.62 (1.37,1.90)	1.27 (1.24,1.73)
+	-	77	1305	590.04	6.26 (4.80,8.15)	3.45 (2.63,4.52)	3.41 (2.60,4.47)
+	+	324	3578	905.54	9.60 (8.03,11.48)	4.79 (3.98,5.77)	4.05 (3.35,4.89)
GBD	Abdominal obesity						
-	-	184	20,599	89.32	1.00 (reference)	1.00 (ref.)	1.00 (ref.)
-	+	615	27,834	220.95	2.48 (2.10,2.92)	1.58 (1.34,1.87)	1.25 (1.04,1.50)
+	-	71	1224	580.07	6.41 (4.87,8.43)	4.41 (3.34,5.81)	4.28 (3.24,5.65)
+	+	330	3659	901.89	10.01 (8.36,12.00)	4.48 (3.69,5.43)	3.30 (2.68,4.06)

Model a was unadjusted

Model b was adjusted for age and sex

Model c was further adjusted for exercise, hypertension, T2DM, overweight, and HDL levels

d Rate per 10,000 person-years. (GBD gallbladder disease, T2DM type 2 diabetes mellitus, CVD cardiovascular disease, HDL high-density lipoprotein cholesterol)

## Supplementary Information

**Additional file 1:** **Supplementary Table S1. **Overall baseline characteristics of the studypopulation for the GBD subgroup. **Supplementary Table S1. ** should appear in the** 4.1 Baseline characteristics **section of thearticle. **Supplementary Figure S1. **Forest plot of the results of the multi-factor Cox regression analysis for CVD. **Supplementary Figure S2**. Kaplan–Meier estimates for cumulative CVDincidence based on the presence of GBD, hypertension, T2DM, dyslipidaemia, overweight, abdominal obesity.

## Data Availability

Some or all datasets generated during and/or analysed during the current study are not publicly available but are available from the corresponding author on reasonable request.
